# Parent and Physician Global Assessment Discordance in Juvenile Arthritis: The Role of Pain Coping Strategies

**DOI:** 10.1016/j.jpedcp.2025.200186

**Published:** 2025-10-10

**Authors:** Maria Backström, Anna Halttu, Maarit Tarkiainen, Ella Lehtinen, Sirja Sard, Paula Keskitalo, Kati Markula-Patjas, Katariina Rebane, Kristiina Aalto, Terhi Remes-Pakarinen, Johanna Kärki, Maiju Hietanen, Heini Pohjankoski, Katja Korkatti, Emmi Kosonen, Eliisa Löyttyniemi, Merja Malin, Liisa Kröger, Paula Vähäsalo

**Affiliations:** 1 Research Unit of Clinical Medicine, University of Oulu, Oulu, Finland; 2 Department of Pediatrics, The Wellbeing Services County of Ostrobothnia, Vaasa, Finland; 3 Medical Research Center, Oulu University Hospital and University of Oulu, Oulu, Finland; 4 Department of Pediatrics, Oulu University Hospital, Oulu, Finland; 5 New Children's Hospital, Helsinki University Hospital, Helsinki, Finland; 6 Pediatric Research Center, University of Helsinki, Helsinki, Finland; 7 Department of Pediatrics, Wellbeing Services of Pirkanmaa, Tampere University Hospital, Tampere, Finland; 8 Faculty of Medicine and Health Technology, Center for Child, Adolescent and Maternal Health Research, Tampere University, Tampere, Finland; 9 Department of Children and Adolescents, Kuopio University Hospital, Kuopio, Finland; 10 Department of Children and Adolescents, Kanta Häme Central Hospital, Hämeenlinna, Finland; 11 The Finnish Rheumatology Quality Register, The Finnish Institute for Welfare and Health, Helsinki, Finland; 12 Department of Pediatrics, Päijät-Häme Central Hospital, Lahti, Finland; 13 Department of Pediatrics, Central Ostrobothnia Central Hospital, Kokkola, Finland; 14 Department of Biostatistics, University of Turku, Turku, Finland; 15 City of Helsinki, Health center, Helsinki, Finland

**Keywords:** catastrophizing, juvenile idiopathic arthritis, outcome measure

## Abstract

**Objective:**

To evaluate the factors underlying the discordance in physicians', patients', and caregivers' perceptions of disease state in patients with juvenile idiopathic arthritis and their caregivers in a prospective observational study.

**Study design:**

We invited all children in 8 centers in Finland from November 2021 to March 2024 presenting with newly confirmed or suspected juvenile idiopathic arthritis and the accompanying parents to participate. Children older than 8.0 years and all the parents completed the patient and parent proxy pain and global assessment of wellbeing at 0 and 3 months and the pain coping scale at 3 months after diagnosis. The discordance between patient or parent global assessment and physician global assessment of disease activity was determined by subtracting the physician global from the patient or parent global assessment. The factors explaining discordance between the global assessments, were evaluated by a multivariable linear model.

**Results:**

In the study, 186 families participated. A positive or negative discordance of 30 mm or greater was seen in 17% of the children and 11% of the caregivers. In the children, the lower the active joint count (AJC) (*P* = .006) and the greater the pain assessment (*P* < .001), the greater the discordance between patient and physician global assessment. In parents, the lower the AJC (*P* < .001) and the higher the parent proxy pain assessment (*P* < .001) and catastrophizing score (*P* < .005), the greater the discordance between parent and physician global assessment.

**Conclusion:**

The discordance between parent's and physician's global assessment is greater among parents who use catastrophizing as a pain coping strategy.

## Graphical abstract

**Figure:**
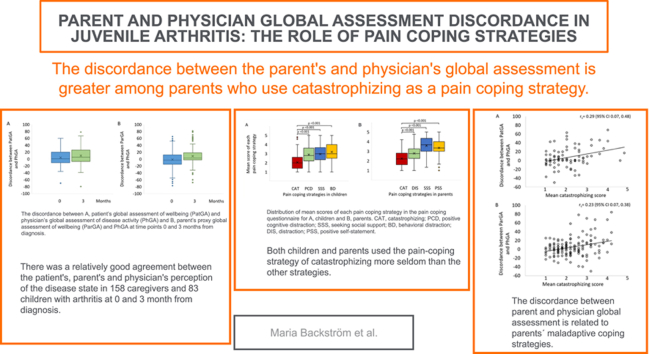

Juvenile idiopathic arthritis (JIA) is diagnosed in children when arthritis is identified in at least 1 joint for 6 weeks or longer.^1^ Disease activity in JIA is measured by Juvenile Arthritis Disease Activity Score, which includes 4 parameters: physician global assessment of disease activity using a 100-mm visual analog scale (PhGA), patient or parent proxy global assessment of well-being (PatGA or ParGA) using a 100-mm visual analog scale, active joint count (AJC), and erythrocyte sedimentation rate level.^2^

It has been shown previously that there can be a discordance between PatGA and PhGA in patients with JIA.^3^, ^4^, ^5^ The factors influencing the discordance have rather extensively been investigated in adults with rheumatoid arthritis (RA).^6^, ^7^, ^8^, ^9^, ^10^ These studies have shown that depression and pain are drivers of positive discordance in patients with RA.^6^^,^^8^, ^9^, ^10^ Limited health literacy also is associated with discordance between PatGA and PhGA.^7^ Only few studies on this subject have been performed in children with JIA.^3^, ^4^, ^5^

Pain is an important parameter influencing the patient's or parent's opinion of the disease activity.^3^, ^4^, ^5^^,^^11^ Despite the clinical remission, a subgroup of patients still experiences pain. The trend occurs partly because of beliefs specific to pain regarding disability and harm as well as the pain-coping strategy of catastrophizing.^12^ Regarding chronic pain, it has been suggested that both the patient's and parent's coping strategies have an essential role in determining outcomes. Coping means cognitive and behavioral actions to overrule the negative impact of stress. Fruitful coping strategies, for instance, include “seeking social support” and “behavioral distraction.” There is also a maladaptive coping strategy, ie, catastrophizing, an exaggerated negative “mental set” during the experience of pain, which has been shown to contribute to more intense pain.^13^

Resilience is an individual's ability to respond effectively to risks or adversities and is a protective factor.^14^ Developmental, social, cultural, and environmental factors influence one's resilience process, including posttraumatic growth, benefit-finding, optimism, and hope. Parental pain coping can be a resilience factor and, at best, protect the child from the decline of functionality.^15^ Parents are crucial in supporting the child's adaptive or maladaptive pain coping,^16^, ^17^, ^18^, ^19^ ie why it is essential to define the coping strategies used by pediatric patients and caregivers.

Recently, we developed a pain-coping questionnaire that enabled us to clarify the pain-coping strategies used by parents when their child is in pain.^19^ We aimed to investigate the discordance in physicians', patients', and caregivers' perceptions of disease state in patients with newly diagnosed or suspected JIA. We will define the factors underlying this discordance and consider the pain-coping strategies patients and parents use.

## Methods

We invited all children from November 2021 to March 2024 presenting with newly confirmed or suspected diagnoses of JIA and the accompanying parents in 8 Finnish hospitals to participate in this prospective observational study. Exclusion criteria were the inability to speak Finnish, Swedish, or English fluently of the parent or child older than 8.0 years. If 2 parents were accompanying the child at the first visit, the parents could decide which of them would contribute. Patients older than 6 years of age and all parents were given written and verbal information and asked to sign a written informed consent form. Consent was obtained according to the declaration of Helsinki. Oulu University Hospital's ethical committee gave the study a positive statement (EETTMK 79/2019). The data were registered at 0 and 3 months from diagnosis of arthritis. The attending children older than 8.0 years and the parents of the children of all ages were asked to fill in patient-reported outcome measures (PROMs) on linear visual analog scales from 0 to 100 mm; the patient's (PatPA) and parent's proxy pain assessment (ParPA), and the PatGA and ParGA at 0 and 3 months from diagnosis and the pain coping scale for children (PCSped) and their parents (PCSpar)^19^ at 3 months from diagnosis. The discordance between the PatGA or ParGA and the PhGA was determined by subtracting the PhGA from the PatGA or ParGA. Hence, the discordance was positive when the PatGA or ParGA was greater than the PhGA and negative when the PatGA or ParGA was smaller than the PhGA. Further registered data were sex, the results of rheumatoid factor levels (twice at least 2 months apart), cyclic citrullinated antibody levels (twice at least 2 months apart), antinuclear antibody levels, HLAB27, duration since the start of symptoms (if exact date was not known, the nearest months first day was recorded), and the presence of comorbidity. Age, ILAR category of JIA, AJC, C-reactive protein, erythrocyte sedimentation rate, PhGA on a linear visual analog scale from 0 to 100 mm, the state of uveitis (the number of cells measured in the worse eye during the most recent slit lamp examination), the date when uveitis was diagnosed for the first time, and data on the synthetic or biologic disease-modifying anti-rheumatic drugs at the visit were recorded both at 0 and 3 months from diagnosis.

Only positive rheumatoid factor results are controlled as part of clinical practice in Finland. Some of the positive results were repeated a little earlier than 3 months after the first positive result. To avoid missing these, we accepted that the second sample was taken at least 2 months apart.

Continuous variables are summarized with medians and lower (Q1) and upper (Q3) quartiles, and categorical variables are reported with counts and percentages. The mean of the points in each pain-coping strategy was calculated. The normal distribution of the data was checked with the Kolmogorov-Smirnov test, and nonparametric tests were used if the data were not normally distributed. The differences between groups were compared using the Mann-Whitney *U* test. The correlations were analyzed using the Spearman correlation. Missing data were handled by pairwise deletion. The factors explaining discordance between PhGA and PatGA or ParGA were evaluated by a multivariable linear model where catastrophizing, sex, children's age, AJC, PatPA or ParPA, comorbidity, and duration of symptoms before diagnosis were entered in the model. To detect a minimum Spearman's rank correlation coefficient (r_s_) of 0.3 with a 2-tailed alpha of 0.05 and a power of 80% we calculated the required sample size of 85. Analyses were performed in SPSS Statistics, version 28.0.0.0 (190) (IBM Corp).

## Results

Of the 186 families participating in the study, at 3 months, 168 of the accompanying caregivers filled in the PCSpar, and 93 of the 104 children who were mature enough to fill in the PCSped completed the questionnaire (Table I). One-third of these children responded to the questionnaires alone or together with the nurse at the pediatric rheumatology outpatient clinic. Regarding the rest of the children, the child had responded to the questionnaire with the caregiver or then the information on whether the child had responded alone or with the caregiver was not provided. The PhGA was available for 167 of 168 children, and the ParGA was available for 158 of 168 children whose caregivers had filled in the PCSpar. The PhGA was available for 93 of 93 children, and PatGA was available for 83 of 93 children who had filled in the PCSped. The reason for missing survey responses was mainly due to oblivion of the healthcare staff.

**Table I:** The PCSped and PCSpar: response frequency (%) and median of the scores

Questions sorted by pain coping strategies	Response frequency, No. (%)	Median (Q1, Q3)
		1 = never, 2 = rarely, 3 = sometimes, 4 = often, 5 = very often
When I am in pain for a couple of hours or days, I…		
Catastrophizing (CATped)		
Q3. Worry that the pain will never stop	92 (49)	2 (1, 3)
Q5. Think all the time how much I am aching	92 (49)	2 (1, 3)
Q7. Think that nothing will help	92 (49)	1.5 (1, 2.75)
Q11. Think that the pain will never ease off	93 (50)	2 (1, 3)
Q15. Worry about my pain almost all the time	91 (49)	2 (1, 2)
Positive cognitive distraction (PCDped)		
Q6. Explain to myself that there is nothing to worry about	93 (50)	2 (1, 3.5)
Q8. Say to myself that soon everything will be all right	93 (50)	2 (1, 4)
Q10. Try not to think about the pain	93 (50)	4 (3, 4)
Q13. Explain to myself that I can overcome anything at all	92 (49)	3 (1.25, 4)
Q14. Do something that will take the pain of my mind	93 (50)	4 (3, 4)
Seeking social support (SSSped)		
Q1. Tell a friend how I feel	93 (50)	3 (2, 4)
Q4. Talk with someone about how I feel	92 (49)	3 (2, 4)
Q12. Talk about my feelings to a friend	93 (50)	3 (2, 4)
Behavioral distraction (BDped)		
Q2. Start doing something	93 (50)	3 (2, 4)
Q9. Start busying myself with something	93 (50)	3 (2, 4)
PCSpar, When my child is in pain for a couple of hours or days I …		
Catastrophizing (CATpar)		
Q3. Worry that my child's pain will never stop	168 (90)	2.5 (2, 4)
Q6. Think all the time how much my child is aching	168 (90)	2 (2, 3)
Q8. Think that nothing will help	168 (90)	2 (1, 2)
Q11. Think that my child's pain will never ease off	168 (90)	2 (1, 2)
Q15. Worry about my child's pain almost all the time	168 (90)	2 (2, 3)
Distraction (DISpar)		
Q2. Start doing something	167 (90)	3 (2, 3)
Q5. Try to focus on something else than my child's pain	168 (90)	3 (2, 4)
Q10. Try not to think about my child's pain	167 (90)	3 (2, 3)
Q14. Do something that will take my child's pain of my mind	167 (90)	3 (2, 3)
Seeking social support (SSSpar)		
Q1. Tell a friend or spouse how I feel	168 (90)	4 (3, 4)
Q4. Talk with someone about how I feel	166 (89)	3 (3, 4)
Q12. Unburden my feelings to a friend or spouse	168 (90)	4 (3, 4)
Positive self-statement (PSSpar)		
Q7. Say to myself there is nothing to worry about my child	167 (90)	3 (3, 4)
Q9. Explain to myself that soon everything will be all right	168 (90)	3 (3, 4)
Q13. Assure myself that we can overcome anything at all	168 (90)	4 (3, 4)

*BD*, behavioral distraction; *CAT*, catastrophizing; *DIS*, distraction; *PCD*, positive cognitive distraction; *PSS*, positive self-statement; *Q1*, 25th percentile; *Q3*, 75th percentile; *SSS*, seeking social support.

**Figure 1:**
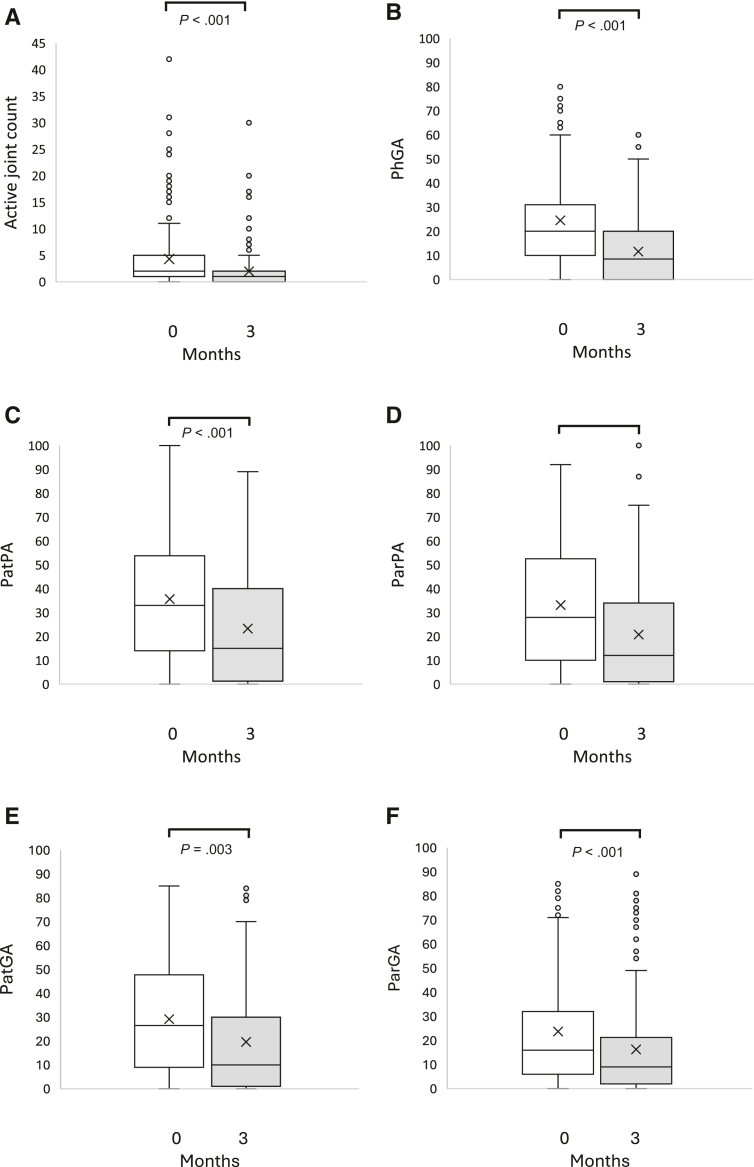
**A,** Active joint count; **B,** PhGA; **C,** PatPA; **D,** ParPA; **E,** PatGA; **F,** ParGA at diagnosis (0 months) and 3 months from diagnosis. The *box* represents Q1, Q3 (IQR), the *line* the median, and the *x* the mean value. The *whiskers* represent minimum and maximum values within 1.5 × IQR, points outside this range are considered outliers. *Q1*, 25th percentile; *Q3*, 75th percentile.

**Figure 2:**
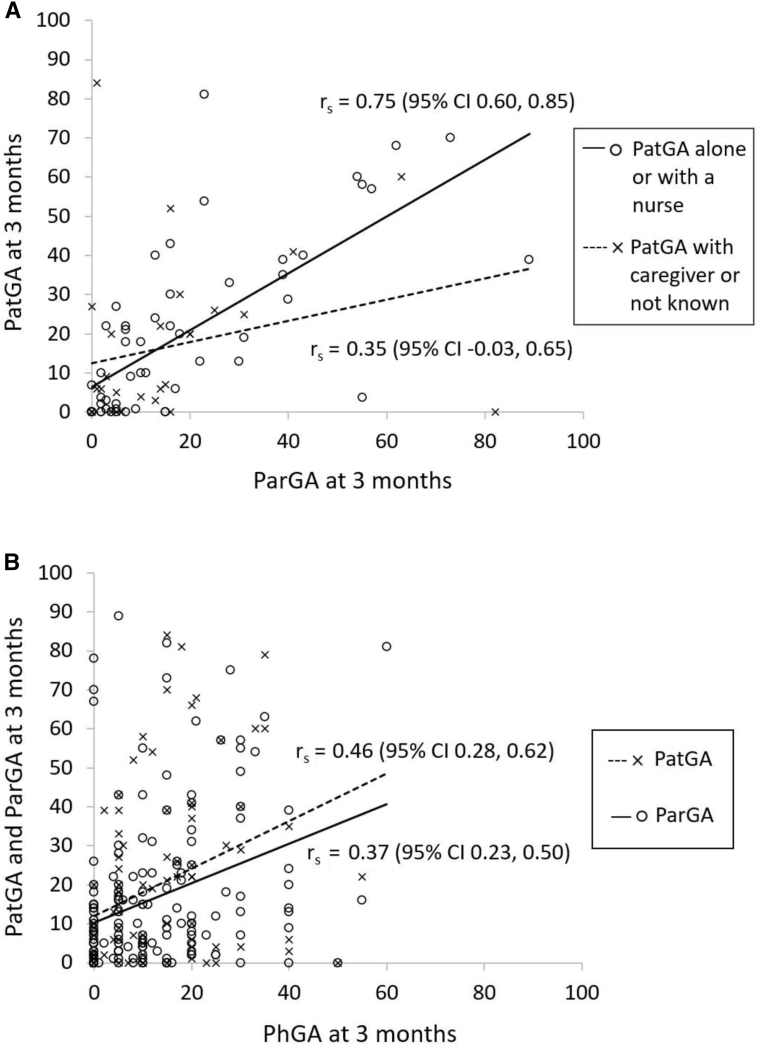
PatGA, ParGA, and PhGA. Spearman correlations at 3 months between **A,** PatGA and ParGA, questionnaires filled alone or with a nurse (*open circles*) or with caregiver or not known (x) and **B,** PatGA, ParGA, and PhGA. *rs*, Spearman rank correlation coefficient.

The median age at diagnosis was 9.0 years, and 55% were girls (Table II). Both the parameters evaluated by the physician (AJC and PhGA) and the PROMs (PatPA, ParPA, PatGA, and ParGA) improved from diagnosis to the visit at 3 months (Figure 1). PatGA correlated to ParGA, and the correlation was surprisingly even stronger if the child had filled in the questionnaires with the nurse or alone (Figure 2).

**Table II:** Baseline characteristics of patients with juvenile arthritis

Characteristics	Value	Total, n = 186
Age, y, median (Q1, Q3)	9 (4.4, 12.6)	186
Female, No. (%)	105 (57)	186
Duration since start of symptoms, mo, median (Q1, Q3)	3.9 (1.9, 7.7)	186
Uveitis at 3 mo, No. (%)	13 (7)	186
Patients on DMARDs at ≤3 mo, No. (%)	145 (78)	186
ANA titer ≥160, No. (%)	95 (51)	186
CCP-ab positive, No. (%)	4 (2)	177
CRP, mg/L, median (Q1, Q3)	3 (2, 13)	157
ESR, mm/h, median (Q1, Q3)	11 (5, 26)	183
HLAB27 positive, No. (%)	56 (30)	184
RF positive, No. (%)	4 (2)	179
Diagnosis (ICD-10), No. (%)		186
M08.0 Juvenile polyarthritis, seropositive	3 (2)	
M08.1 Entesitis related juvenile arthritis	12 (7)	
M08.2 Systemic juvenile arthritis	1 (0.5)	
M08.3 Juvenile polyarthritis, seronegative	27 (15)	
M08.8 Other juvenile arthritis	5 (3)	
M08.9 Unspecified juvenile idiopathic arthritis	117 (63)	
M09.0∗ L40.5 Juvenile psoriatic arthritis	1 (0.5)	
M13.0 Unspecified polyarthritis	1 (0.5)	
M13.9 Unspecified arthritis	17 (9)	
M13.9, L50.9 Urticaria arthritis	1 (0.5)	
K07.6 Temporomandibular joint disorder	1 (0.5)	

*ANA*, antinuclear antibodies; *CCP-ab*, cyclic citrullinated peptide antibodies; *CRP*, C-reactive protein; *ESR*, erythrocyte sedimentation rate; *DMARDs*, disease-modifying antirheumatic drugs; *HLAB27*, Human leukocyte antigen B27; *ICD-10*, *International Classification of Diseases*, *Tenth Revision*; *RF*, rheumatoid factor.

**Figure 3:**
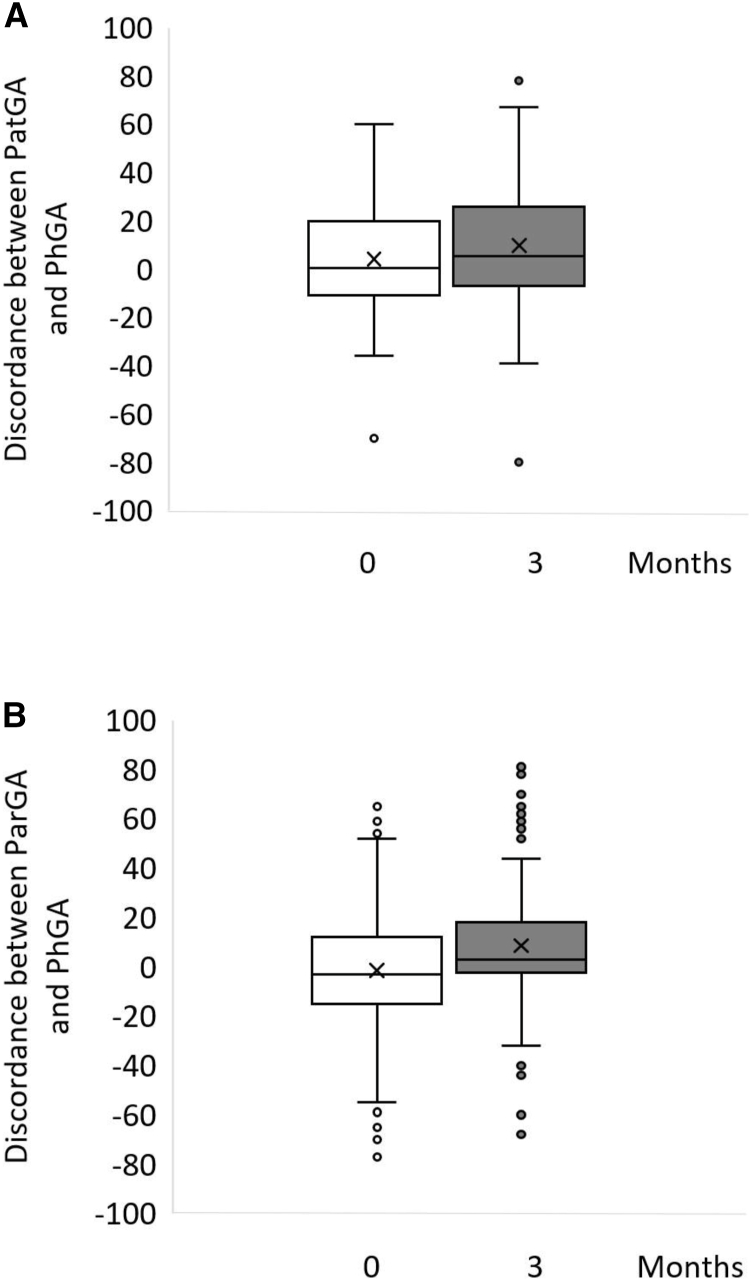
PatGA, ParGA, and PhGA discordance. The discordance between **A,** PatGA and PhGA and **B,** ParGA and PhGA at time points 0 and 3 months from diagnosis, determined by subtracting the PhGA from PatGA and ParGA. The *box* represents Q1, Q3 (IQR), the *line* the median, and the *x* the mean value. The *whiskers* represent minimum and maximum values within 1.5 × IQR, points outside this range are considered outliers.

The PhGA correlated significantly to the PatGA and ParGA at 3 months (Figure 2), but in some families, there was an apparent discordance between PatGA or ParGA and PhGA (Figure 3). The mean (SD) discordance between PatGA and PhGA was 7.0 (21.5) mm, and between ParGA and PhGA, 4.5 (20.1) mm. A positive or negative discordance of less than 10 mm was seen in 51% of the children and 52% of the caregivers. When a discordance of more than 10 mm was seen, it was mainly positive, ie, the patients' and parents' perception of the disease state was worse than the physician's. Only 17% of the children and 19% of the caregivers rated the global assessment at least 10 mm lower than the physician. A positive discordance of 10 mm or greater was seen in 35% of the children and 32% of the caregivers, and a positive discordance of 30 mm or greater was seen in 13% of the children and 8% of the caregivers. A positive or negative discordance of 30 mm or greater was seen in 17% of the children and 11% of the caregivers. In the children, the discordance was greater in girls than in boys (median 9 vs 0, *P* = .039).

**Figure 4:**
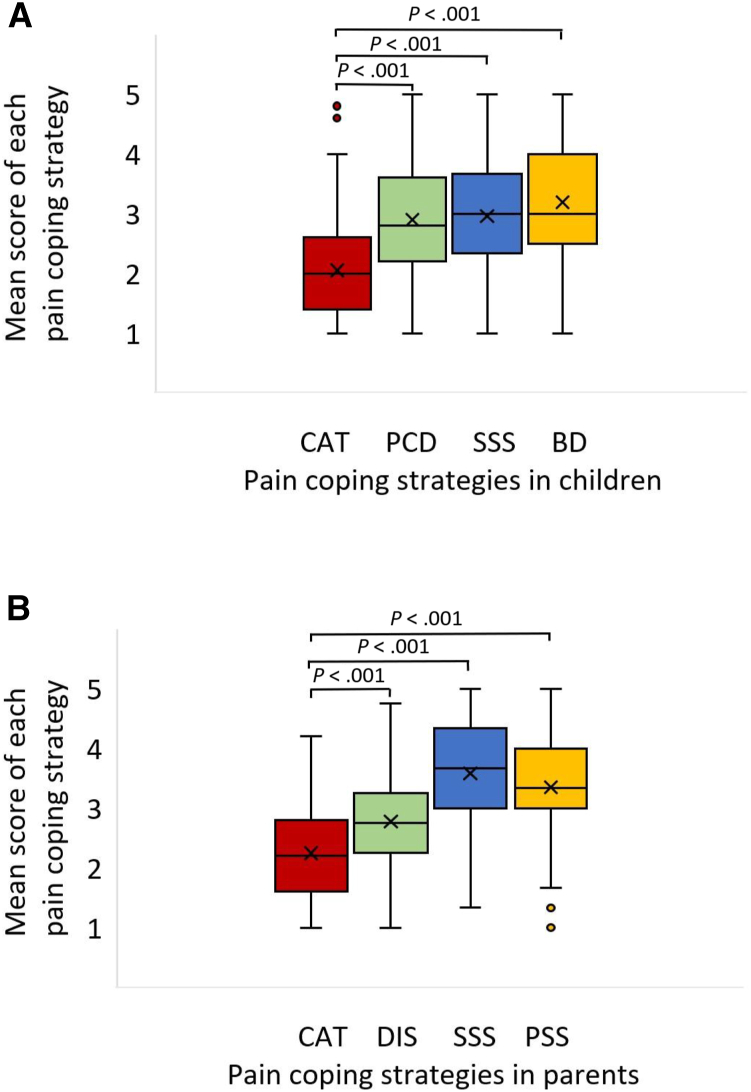
Distribution of mean scores of each pain coping strategy in the pain coping questionnaire. Distribution of mean scores of each pain coping strategy in the pain coping questionnaire for **A,** children and **B,** parents. The *box* represents Q1, Q3 (IQR), the *line* the median, and the *x* the mean value. The *whiskers* represent minimum and maximum values within 1.5 × IQR, points outside this range are considered outliers. *BD*, behavioral distraction; *CAT*, catastrophizing; *DIS*, distraction; *PCD*, positive cognitive distraction; *PSS*, positive self-statement; *SSS*, seeking social support.

Both children and parents used the pain-coping strategy of catastrophizing more seldom than the other strategies (Figure 4). The most used strategies were behavioral distraction in children and seeking social support in caregivers.

**Figure 5:**
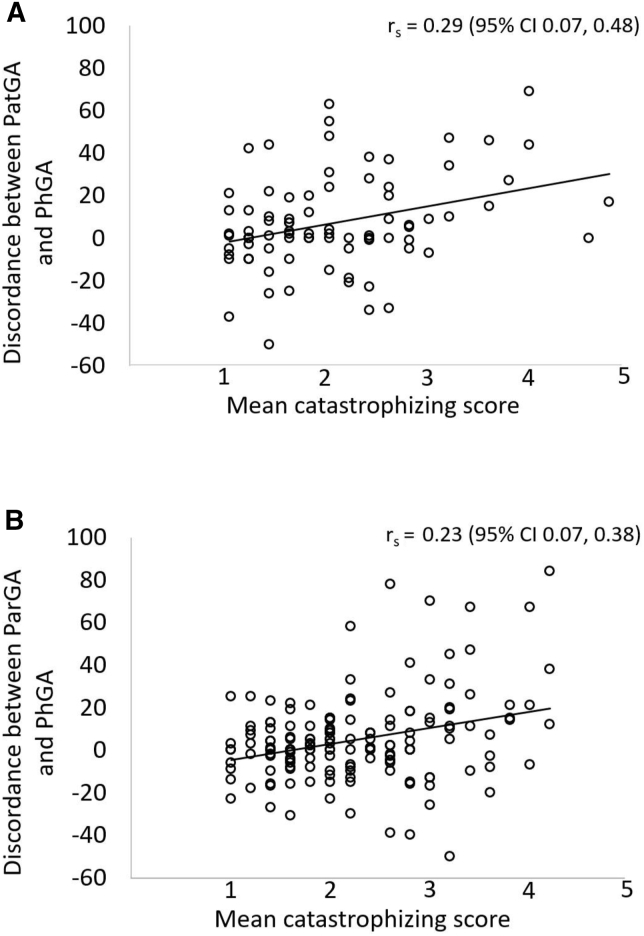
PatGA, ParGA, and PhGA discordance and the mean catastrophizing score. Spearman correlation between the discordance of **A,** PatGA and PhGA and **B,** ParGA and PhGA and the mean catastrophizing score.

There was a significant, positive correlation between the mean value for patients' and caregivers' catastrophizing and the discordance between PatGA or ParGA and PhGA, ie, when the patient or caregivers had a greater catastrophizing score, a greater discordance occurred between PatGA or ParGA and PhGA (Figure 5). There was also a positive correlation between parents' and patients' catastrophizing scores (r_s_ = 0.30; 95% CI 0.10-0.48). The question in the PCSped that had the strongest correlation to discordance between PatGA and PhGA was question number 11: “I think that the pain will never ease off” (r_s_ = 0.33; 95% CI 0.12-0.51). In PCSpar question number 8:”I think that nothing will help” had the strongest correlation to discordance between ParGA and PhGA (r_s_ = 0.36; 95% CI 0.22-0.49).

For the children, the catastrophizing score was no longer significant when considering the other possible drivers of PatGA and PhGA discordance using a multivariable linear model. The main drivers of discordance for the children were AJC (*P* = .006) and PatPA (*P* < .001), that is, the lower the AJC and the greater the PatPA, the greater the differences between PatGA and PhGA. Regarding the caregivers, the drivers of ParGA and PhGA discordance were catastrophizing score (*P* = .005), ParPA (*P* < .001), and AJC (*P* < .001), ie, the lower the AJC and the greater the ParPA and catastrophizing score, the greater was the discordance. The mean of the other pain coping strategies did not correlate to PatPA, ParPA, AJC nor with the discordance between PatGA or ParGA and PhGA at 3 months.

## Discussion

We have presented the coping strategies of children with JIA and provided novel insight into parental coping strategies when their child is newly diagnosed with arthritis. In line with earlier studies, both children and caregivers in our study used effective coping mechanisms for adjusting to the new JIA diagnosis more often than maladaptive coping strategies, ie catastrophizing.^13^ For the first time, we have shown that the discordance between PatGA or ParGA and PhGA is related to catastrophizing. In agreement with earlier reports,^3^^,^^4^ other drivers of discordance between PatGA or ParGA and PhGA were pain and disease activity, as judged by AJC in this study. The greater the pain score and the lower the AJC, the greater was the discordance. When other possible drivers were considered by a linear model, catastrophizing, pain, and AJC remained in the model for the caregivers but only pain and AJC for the children. This finding is in line with earlier reports done in adults with RA.^6^^,^^8^, ^9^, ^10^ A positive or negative discordance less than 10 mm was seen in 51% of the children and 52% of the caregivers, a slightly better result than in earlier JIA studies from Italy, where a positive or negative discordance less than 10 mm was seen in 40% of the caregivers.^3^^,^^4^ When discordance was seen in this study, it was mainly positive, ie, the patients and parents rated the disease state greater than the physician, which is in sharp contrast with an earlier JIA study, where the discordance was mainly negative,^3^ but in agreement with studies in adults with RA, in which the discordance has usually been positive.^6^, ^7^, ^8^, ^9^ The fact that the study is conducted early in the disease can contribute to a mainly positive discordance, because the disease is active and the pain is more prominent. This would be logical because in our study, pain is a driver of a positive discordance. A positive or negative discordance of 30 mm or greater was seen in only 17% of the children and 11% of the caregivers, contrary to earlier studies where the corresponding numbers were 34% in JIA^3^ and 36% in a systematic literature review regarding RA.^9^

PROMs are meaningful additions to the physician's evaluation of the patient, providing a broader picture of the impact of JIA on the patient and enabling more individualized therapy. It has been shown that PatGA increases at the visit before a flare, while the PhGA scores do not,^5^ indicating a predictive value of PatGA that PhGA does not have. The global assessment might be interpreted differently by patients and physicians. Patients' and parents' evaluation may reflect disease impact, not just the pathologic severity implied by disease activity. However, an apparent discordance between PatGA or ParGA and PhGA may impact shared decision-making. Therefore, efforts to recognize the drivers behind and interventions to reduce the discordance are needed. JIA patients with effective pain coping strategies have less pain.^13^ In our study, we showed that the caregiver's and child's use of maladaptive pain coping strategies correlated, and that the caregivers' catastrophizing was related to the discordance between ParGA and PhGA. Our results highlight the importance of surveying not only the children's but also the caregiver's pain coping strategies at the JIA visit. In clinical practice, completing PCSped and PCSpar is swift and feasible.^20^ On the basis of the survey results, a collaborative approach between the patient, the physician, and other health professionals can support patients and caregivers towards effective coping strategies when needed. Such support could ease the caregiver's burden and help managing perceptions of illness and coping with pain-related distress.

The strengths of this study are that both children's and caregivers' perspectives have been studied in a multicentre prospective setting and the inclusion of all non-systemic JIA categories shortly after diagnosis. However, there also are limitations of our study. Only one-third of the children answered the questionnaires alone or with the nurse at the pediatric rheumatology outpatient clinic. Regarding the rest of the children, the information on whether the child had responded alone or with the caregiver was not provided. Moreover, the data were not complete in the whole data set. This can have an impact on the results. Further, we have not been able to identify or investigate the full spectrum of the potential explanatory factors of discordance, eg, health expectations, personal life events, and the quality of interaction between the patient and the physician. This multicenter study was conducted in a large part of Finland but was not international. Hence, environmental and cultural factors might alter the result in a more extensive study. Every patient did not have a JIA diagnosis at the time of our study. *The International Classification of Diseases*, *Tenth Revision*, codes M13.0 and M13.9 are sometimes used in Finland during the first visits, changing to M08 later when the diagnosis is verified. Therefore, some of the patients who were coded as M13 may be, in fact, patients with JIA. There are only 2 patients for sure who do not have JIA: the patient with urticaria arthritis and the patient with temporomandibular joint disorder. Removing these 2 patients from the analyses does not change the results, but we cannot be sure that these results can be generalized to JIA populations.

Given the impact of JIA on quality of life can have an alteration over time, and the duration of the study was only from 0 to 3 months after JIA diagnosis, there may be changes in discordance and pain-coping strategies in a longer perspective. The coping strategies at 3 months of disease duration can be significantly different from those after a longer disease duration, when the patient and parent have become accustomed to living with the chronic disease. In the future, we will report data from the 1- and 2-year follow-up of these patients. Moreover, international longitudinal assessments of discordance and its relation to pain coping strategies are needed.

In conclusion, we showed for the first time that the greater the parent's catastrophizing score, the greater the discordance between the parent's and physician's global assessment. The discordance between the patient's or parent's and physician's global assessment was smaller than in earlier studies.^3^^,^^9^ Children and caregivers used effective pain coping strategies more often than catastrophizing. Screening pain coping strategies as a part of routine clinical care is vital to detect those needing patient empowerment, education, and support in using constructive coping strategies to strengthen their resilience. Further studies are required to determine whether such support would diminish the discordance between patient or parent and physician global assessment.

## CRediT authorship contribution statement

**Maria Backström:** Writing – original draft, Visualization, Validation, Software, Resources, Project administration, Methodology, Investigation, Funding acquisition, Formal analysis, Data curation, Conceptualization. **Anna Halttu:** Writing – original draft, Visualization, Resources, Investigation, Formal analysis. **Maarit Tarkiainen:** Writing – review & editing, Validation, Resources, Methodology, Investigation, Conceptualization. **Ella Lehtinen:** Writing – review & editing, Writing – original draft, Resources. **Sirja Sard:** Writing – review & editing, Validation, Resources, Methodology, Investigation, Conceptualization. **Paula Keskitalo:** Writing – review & editing, Resources, Methodology, Investigation, Conceptualization. **Kati Markula-Patjas:** Writing – review & editing, Validation, Resources, Methodology, Investigation, Conceptualization. **Katariina Rebane:** Writing – review & editing, Validation, Resources, Methodology, Investigation, Conceptualization. **Kristiina Aalto:** Writing – review & editing, Validation, Resources, Methodology, Investigation, Conceptualization. **Terhi Remes-Pakarinen:** Writing – review & editing, Validation, Resources, Methodology, Investigation, Conceptualization. **Johanna Kärki:** Writing – review & editing, Validation, Resources, Methodology, Investigation, Conceptualization. **Maiju Hietanen:** Writing – review & editing, Validation, Resources, Methodology, Investigation, Conceptualization. **Heini Pohjankoski:** Writing – review & editing, Validation, Resources, Methodology, Investigation, Data curation, Conceptualization. **Katja Korkatti:** Writing – review & editing, Validation, Resources, Methodology, Investigation, Conceptualization. **Emmi Kosonen:** Writing – review & editing, Resources, Investigation. **Eliisa Löyttyniemi:** Writing – review & editing, Writing – original draft, Software, Methodology, Formal analysis. **Merja Malin:** Writing – review & editing, Validation, Resources, Methodology, Investigation, Conceptualization. **Liisa Kröger:** Writing – review & editing, Validation, Resources, Methodology, Investigation, Conceptualization. **Paula Vähäsalo:** Writing – review & editing, Visualization, Validation, Supervision, Resources, Project administration, Methodology, Investigation, Funding acquisition, Conceptualization.

## Data Statement

Data sharing statement available at www.jpeds.com.

## Declaration of Competing Interest

The authors declare that they have no known competing financial interests or personal relationships that could have appeared to influence the work reported in this paper.

## Supplementary material

Data Statement
