# Supplementary material for: Parent and Physician Global Assessment Discordance in Juvenile Arthritis: The Role of Pain Coping Strategies
Source: J Pediatr Clin Pract. 2025 Oct 10;18:200186. doi: 10.1016/j.jpedcp.2025.200186 (PMC12766085; doi:10.1016/j.jpedcp.2025.200186)
Supplement: Data Statement [file mmc1.docx]

Data Statement

Data are available from the corresponding author upon reasonable request.
